# Aqua­[2-(3-carb­oxy-5-carboxyl­atophen­oxy)acetato-κ*O* ^1^]bis­(1,10-phenanthroline-κ^2^ *N*,*N*′)manganese(II) dihydrate

**DOI:** 10.1107/S1600536810048142

**Published:** 2010-11-24

**Authors:** Yi-Ni Cai, Jing Chen, Yun-Long Feng

**Affiliations:** a Zhejiang Key Laboratory for Reactive Chemistry on Solid Surfaces, College of Chemistry and Life Science, Zhejiang Normal University, Jinhua, Zhejiang 321004, People’s Republic of China

## Abstract

In the title complex, [Mn(C_10_H_6_O_7_)(C_12_H_8_N_2_)_2_(H_2_O)]·2H_2_O, the Mn^II^ atom is coordinated by two O atoms from one 2-(3-carb­oxy-5-carboxyl­atophen­oxy)acetate (HOABDC^2−^) dianion and one water mol­ecule and by four N atoms from two 1,10-phenanthroline (phen) ligands within a distorted octa­hedral geometry. O—H⋯O hydrogen bonding between –COOH and –COO^−^ groups of adjacent mol­ecules and between carboxyl­ate groups and coordinated and uncoordin­ated water mol­ecules leads to a three-dimensional structure which is further stabilized by weak π–π inter­actions of adjacent phen ligands with centroid–centroid separations of 4.2932 (1) Å.

## Related literature

For related structures, see: Cao *et al.* (2004 ▶, 2007 ▶); Cheng *et al.* (2004 ▶); Murugavel *et al.* (2002 ▶); Zhang *et al.* (2002 ▶).
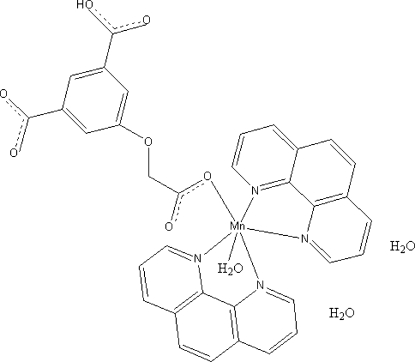


## Experimental

### 

#### Crystal data

[Mn(C_10_H_6_O_7_)(C_12_H_8_N_2_)_2_(H_2_O)]·2H_2_O
*M* *_r_* = 707.54
Monoclinic, 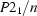

*a* = 8.1024 (2) Å
*b* = 22.3106 (6) Å
*c* = 17.5381 (5) Å
β = 99.212 (2)°
*V* = 3129.46 (14) Å^3^
*Z* = 4
Mo *K*α radiation
μ = 0.49 mm^−1^
*T* = 296 K
0.29 × 0.24 × 0.10 mm

#### Data collection

Bruker APEXII CCD diffractometer
Absorption correction: multi-scan (*SADABS*; Sheldrick, 1996 ▶) *T*_min_ = 0.871, *T*_max_ = 0.955
69826 measured reflections
7282 independent reflections
5127 reflections with *I* > 2σ(*I*)
*R*_int_ = 0.050

#### Refinement

*R*[*F* ^2^ > 2σ(*F* ^2^)] = 0.038
*wR*(*F* ^2^) = 0.117
*S* = 1.00
7282 reflections
463 parameters
10 restraints
H atoms treated by a mixture of independent and constrained refinement
Δρ_max_ = 0.41 e Å^−3^
Δρ_min_ = −0.26 e Å^−3^

### 

Data collection: *APEX2* (Bruker, 2006 ▶); cell refinement: *SAINT* (Bruker, 2006 ▶); data reduction: *SAINT*; program(s) used to solve structure: *SHELXS97* (Sheldrick, 2008 ▶); program(s) used to refine structure: *SHELXL97* (Sheldrick, 2008 ▶); molecular graphics: *SHELXTL* (Sheldrick, 2008 ▶) and *DIAMOND* (Brandenburg, 2007 ▶); software used to prepare material for publication: *SHELXTL*.

## Supplementary Material

Crystal structure: contains datablocks I, global. DOI: 10.1107/S1600536810048142/wm2428sup1.cif

Structure factors: contains datablocks I. DOI: 10.1107/S1600536810048142/wm2428Isup2.hkl

Additional supplementary materials:  crystallographic information; 3D view; checkCIF report

## Figures and Tables

**Table 1:** Selected bond lengths (Å)

Mn—O6	2.1352 (13)
Mn—O1*W*	2.1820 (15)
Mn—N1	2.2462 (17)
Mn—N4	2.2633 (16)
Mn—N2	2.2776 (16)
Mn—N3	2.2974 (16)

**Table 2:** Hydrogen-bond geometry (Å, °)

*D*—H⋯*A*	*D*—H	H⋯*A*	*D*⋯*A*	*D*—H⋯*A*
O1*W*—H1*WA*⋯O4^i^	0.83 (2)	1.92 (2)	2.740 (2)	174 (2)
O1—H⋯O3^ii^	0.87 (2)	1.56 (2)	2.4148 (19)	169 (3)
O2*W*—H2*WB*⋯O4^iii^	0.84 (2)	2.08 (2)	2.917 (2)	176 (3)
O2*W*—H2*WA*⋯O2^iv^	0.83 (2)	2.08 (2)	2.908 (3)	177 (3)
O3*W*—H3*WB*⋯O3^v^	0.83 (2)	2.55 (2)	3.323 (3)	155 (4)
O3*W*—H3*WA*⋯O2*W*^vi^	0.84 (2)	2.01 (2)	2.845 (3)	173 (5)

Symmetry codes: (i) 
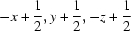
; (ii) 
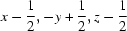
; (iii) 
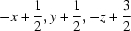
; (iv) 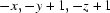; (v) 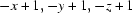; (vi) 
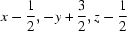
.

**Fig. 1.**
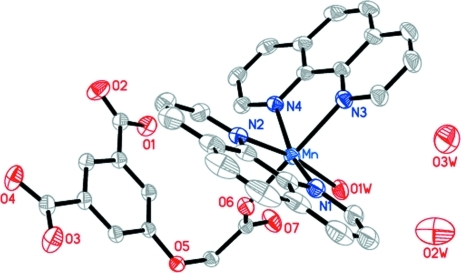
The molecular structure of the title compound. Displacement ellipsoids are drawn at the 30% probability level.

**Fig. 2.**
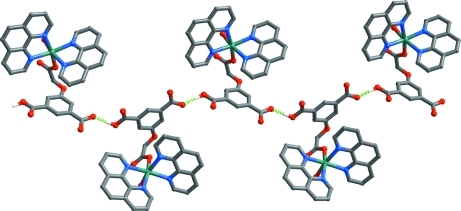
The "T"-shaped chain in the title compound.

**Fig. 3.**
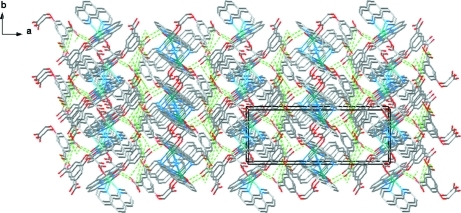
The three-dimensional framework of the title compound with hydrogen bonding and π–π interactions (dashed lines).

## supplementary crystallographic information

### Comment

Many aromatic polycarboxylate ligands have been employed as organic skeletons in the formation of various metal-organic compounds. To the best of our knowledge, aromatic polycarboxylate ligand with both rigid and flexible carboxyl groups are less reported. In comparison with rigid aromatic multicarboxylic ligands (Cheng *et al.*, 2004; Murugavel *et al.*, 2002; Zhang *et al.*, 2002), 5-oxyacetic-1,3-benzenebiscarboxylic acid (H_3_OABDC) provides two rigid carboxyl groups and one flexible oxyacetato group (Cao *et al.*, 2004, 2007). Herein, we report the synthesis and structure of a new Mn^II^ compound based on the H_3_OABDC ligand, [Mn(C_10_H_6_O_7_)(C_12_H_8_N_2_)_2_(H_2_O)]^.^2H_2_O, (I).

Compound (I) consists of one Mn^II^ atom, one HOABDC^2-^ dianion, two phenanthroline (phen) ligands, one coordinated and two lattice water molecules, as shown in Fig. 1. The Mn^II^ atom is six-coordinated by four nitrogen atoms from two phen ligands (Mn—N 2.2462 (17)–2.2974 (16) Å), one oxygen atom from one flexible carboxyl group of the HOABDC^2-^ anion (Mn—O 2.1352 (13) Å) and one water molecule (Mn—O 2.1820 (15) Å) in a distorted octahedral geometry. It is notable that only the flexible carboxylato group participates in a monodentately coordinating mode to the Mn^II^ atom. The dianion is not planar as indicated by the dihedral angles (8.2 (1)–10.17 (5) °) between the benzene ring and the two carboxy groups, as well as the torsion angle (69.0 (2) °) involving the benzene ring and the OCH_2_COO_2_ group. The complex molecules are connected to each other through O—H···O interactions between the two rigid carboxyl groups of the HOABDC^2-^ dianion to form a "T"-shaped chain (Fig. 2). Adjacent chains are further linked with each other *via* weak π–π interactions between phen groups (centroid—centroid separation between planes is 4.2932 (1) Å) and O—H···O hydrogen bonds involving both coordinated and uncoordinated water molecules (Fig. 3).

### Experimental

All reagents were of analytical reagent grade and were used without further purification. A mixture of H_3_OABDC (0.1205 g, 0.5 mmol), Mn(NO_3_)_2_^.^6H_2_O (0.0976 g, 0.34 mmol), phen (0.1986 g, 0.01 mmol), and NaOH (0.0101 g, 0.25 mmol) was dissolved in purified water (15 ml), and reacted in a 25 ml stainless steel reactor with a telflon liner and heated at 433 K for 72 h, and then cooled to room temperature over 3 days. Then, the reactor was cooled to room temperature at a speed of 5 K^.^h^-1^. Yellow single crystals of title compound were obtained by slow evaporation of the filtrate over a few days (yield 40.1% based on H_3_OABDC).

### Refinement

The carbon-bound H-atoms were positioned geometrically and included in the refinement using a riding model [C—H 0.93 Å *U*_iso_(H) = 1.2*U*_eq_(C)]. The oxygen-bound H-atoms were located in difference Fourier maps and were refined with the O—H distances restrained to 0.82 Å [*U*_iso_(H) = 1.2*U*_eq_(O)].

### Crystal data

**Table:**

[Mn(C_10_H_6_O_7_)(C_12_H_8_N_2_)_2_(H_2_O)]·2H_2_O	*F*(000) = 1460
*M**_r_* = 707.54	*D*_x_ = 1.502 Mg m^−^^3^
Monoclinic, *P*2_1_/*n*	Mo *K*α radiation, λ = 0.71073 Å
Hall symbol: -P 2yn	Cell parameters from 9990 reflections
*a* = 8.1024 (2) Å	θ = 1.5–27.7°
*b* = 22.3106 (6) Å	µ = 0.49 mm^−^^1^
*c* = 17.5381 (5) Å	*T* = 296 K
β = 99.212 (2)°	Block, yellow
*V* = 3129.46 (14) Å^3^	0.29 × 0.24 × 0.10 mm
*Z* = 4	

### Data collection

**Table:**

Bruker APEXII CCD diffractometer	7282 independent reflections
Radiation source: fine-focus sealed tube	5127 reflections with *I* > 2σ(*I*)
graphite	*R*_int_ = 0.050
ω scans	θ_max_ = 27.7°, θ_min_ = 1.5°
Absorption correction: multi-scan (*SADABS*; Sheldrick, 1996)	*h* = −10→10
*T*_min_ = 0.871, *T*_max_ = 0.955	*k* = −28→29
69826 measured reflections	*l* = −22→21

### Refinement

**Table:**

Refinement on *F*^2^	Primary atom site location: structure-invariant direct methods
Least-squares matrix: full	Secondary atom site location: difference Fourier map
*R*[*F*^2^ > 2σ(*F*^2^)] = 0.038	Hydrogen site location: inferred from neighbouring sites
*wR*(*F*^2^) = 0.117	H atoms treated by a mixture of independent and constrained refinement
*S* = 1.00	*w* = 1/[σ^2^(*F*_o_^2^) + (0.0638*P*)^2^ + 0.5366*P*] where *P* = (*F*_o_^2^ + 2*F*_c_^2^)/3
7282 reflections	(Δ/σ)_max_ = 0.001
463 parameters	Δρ_max_ = 0.41 e Å^−^^3^
10 restraints	Δρ_min_ = −0.26 e Å^−^^3^

### Special details

**Table:**

Geometry. All e.s.d.'s (except the e.s.d. in the dihedral angle between two l.s. planes) are estimated using the full covariance matrix. The cell e.s.d.'s are taken into account individually in the estimation of e.s.d.'s in distances, angles and torsion angles; correlations between e.s.d.'s in cell parameters are only used when they are defined by crystal symmetry. An approximate (isotropic) treatment of cell e.s.d.'s is used for estimating e.s.d.'s involving l.s. planes.
Refinement. Refinement of *F*^2^ against ALL reflections. The weighted *R*-factor *wR* and goodness of fit *S* are based on *F*^2^, conventional *R*-factors *R* are based on *F*, with *F* set to zero for negative *F*^2^. The threshold expression of *F*^2^ > σ(*F*^2^) is used only for calculating *R*-factors(gt) *etc*. and is not relevant to the choice of reflections for refinement. *R*-factors based on *F*^2^ are statistically about twice as large as those based on *F*, and *R*- factors based on ALL data will be even larger.

### Fractional atomic coordinates and isotropic or equivalent isotropic displacement parameters (Å^2^)

**Table:**

	*x*	*y*	*z*	*U*_iso_*/*U*_eq_	
Mn	0.20775 (3)	0.527200 (13)	0.259979 (16)	0.03977 (10)	
O1W	0.3442 (2)	0.58989 (7)	0.19761 (9)	0.0548 (4)	
H1WA	0.302 (3)	0.6150 (9)	0.1656 (12)	0.066*	
H1WB	0.395 (3)	0.5651 (9)	0.1744 (13)	0.066*	
O1	0.1064 (2)	0.31086 (7)	0.08413 (8)	0.0585 (4)	
H	0.050 (3)	0.2960 (10)	0.0421 (11)	0.070*	
O2W	0.3384 (2)	0.69978 (11)	0.94327 (11)	0.0845 (6)	
H2WB	0.310 (4)	0.6916 (14)	0.9859 (11)	0.101*	
H2WA	0.254 (3)	0.7136 (14)	0.9152 (14)	0.101*	
O2	−0.0390 (2)	0.25197 (9)	0.15014 (9)	0.0759 (5)	
O3W	0.1249 (3)	0.72517 (11)	0.4755 (2)	0.1336 (11)	
H3WB	0.209 (3)	0.7467 (16)	0.486 (3)	0.160*	
H3WA	0.042 (3)	0.7476 (16)	0.462 (3)	0.160*	
O3	0.4840 (2)	0.23215 (7)	0.46173 (8)	0.0724 (5)	
O4	0.2717 (2)	0.17586 (7)	0.40925 (8)	0.0608 (4)	
O5	0.62133 (16)	0.36920 (6)	0.26636 (7)	0.0440 (3)	
O6	0.40064 (17)	0.46344 (6)	0.25174 (8)	0.0455 (3)	
O7	0.4781 (2)	0.49201 (7)	0.14154 (9)	0.0671 (5)	
N1	0.3104 (2)	0.56296 (8)	0.37776 (10)	0.0517 (4)	
N2	0.1241 (2)	0.46239 (7)	0.34659 (9)	0.0444 (4)	
N3	−0.0037 (2)	0.59680 (7)	0.24904 (10)	0.0474 (4)	
N4	0.01674 (19)	0.49978 (7)	0.15726 (9)	0.0424 (4)	
C1	0.2283 (2)	0.28589 (8)	0.21010 (10)	0.0395 (4)	
C2	0.2303 (2)	0.25059 (8)	0.27511 (10)	0.0415 (4)	
H2	0.1429	0.2243	0.2786	0.050*	
C3	0.3647 (2)	0.25495 (8)	0.33519 (10)	0.0387 (4)	
C4	0.4927 (2)	0.29499 (8)	0.32972 (10)	0.0385 (4)	
H4	0.5824	0.2978	0.3699	0.046*	
C5	0.4888 (2)	0.33098 (8)	0.26510 (10)	0.0366 (4)	
C6	0.3576 (2)	0.32568 (8)	0.20429 (10)	0.0387 (4)	
H6	0.3560	0.3486	0.1599	0.046*	
C7	0.0849 (3)	0.28103 (9)	0.14504 (11)	0.0441 (4)	
C8	0.3719 (3)	0.21739 (9)	0.40661 (10)	0.0452 (5)	
C9	0.6173 (2)	0.40997 (9)	0.20347 (11)	0.0436 (4)	
H9B	0.5972	0.3873	0.1557	0.052*	
H9A	0.7266	0.4285	0.2069	0.052*	
C10	0.4870 (2)	0.45928 (8)	0.19925 (11)	0.0391 (4)	
C11	0.4049 (3)	0.61143 (11)	0.39264 (16)	0.0699 (7)	
H11	0.4292	0.6342	0.3514	0.084*	
C12	0.4691 (4)	0.62961 (14)	0.4676 (2)	0.0944 (11)	
H12	0.5327	0.6644	0.4761	0.113*	
C13	0.4381 (4)	0.59635 (16)	0.5274 (2)	0.0949 (12)	
H13	0.4841	0.6074	0.5775	0.114*	
C14	0.3372 (3)	0.54517 (13)	0.51546 (14)	0.0710 (8)	
C15	0.2971 (4)	0.50771 (19)	0.57607 (15)	0.0959 (12)	
H15	0.3358	0.5179	0.6272	0.115*	
C16	0.2060 (4)	0.45879 (19)	0.56048 (16)	0.0913 (11)	
H16	0.1819	0.4354	0.6012	0.110*	
C17	0.1430 (3)	0.44057 (14)	0.48282 (14)	0.0667 (7)	
C18	0.0533 (3)	0.38851 (15)	0.46418 (18)	0.0842 (9)	
H18	0.0275	0.3638	0.5033	0.101*	
C19	0.0027 (3)	0.37328 (13)	0.38935 (19)	0.0801 (8)	
H19	−0.0560	0.3379	0.3767	0.096*	
C20	0.0403 (3)	0.41167 (11)	0.33149 (14)	0.0596 (6)	
H20	0.0050	0.4011	0.2802	0.072*	
C21	0.1775 (2)	0.47682 (10)	0.42138 (11)	0.0482 (5)	
C22	0.2753 (3)	0.52968 (10)	0.43802 (12)	0.0521 (5)	
C23	−0.0184 (3)	0.64320 (10)	0.29509 (14)	0.0587 (6)	
H23	0.0592	0.6470	0.3400	0.070*	
C24	−0.1426 (3)	0.68609 (11)	0.27993 (18)	0.0715 (7)	
H24	−0.1510	0.7168	0.3150	0.086*	
C25	−0.2517 (3)	0.68242 (11)	0.21291 (19)	0.0729 (8)	
H25	−0.3346	0.7113	0.2013	0.088*	
C26	−0.2408 (3)	0.63540 (10)	0.16083 (14)	0.0585 (6)	
C27	−0.3498 (3)	0.62863 (13)	0.08932 (17)	0.0724 (8)	
H27	−0.4302	0.6578	0.0740	0.087*	
C28	−0.3393 (3)	0.58124 (14)	0.04358 (15)	0.0701 (7)	
H28	−0.4115	0.5784	−0.0032	0.084*	
C29	−0.2185 (3)	0.53454 (11)	0.06531 (12)	0.0531 (5)	
C30	−0.2098 (3)	0.48226 (12)	0.02292 (13)	0.0603 (6)	
H30	−0.2854	0.4761	−0.0222	0.072*	
C31	−0.0914 (3)	0.44008 (11)	0.04712 (12)	0.0567 (6)	
H31	−0.0862	0.4048	0.0194	0.068*	
C32	0.0217 (3)	0.45103 (10)	0.11419 (12)	0.0487 (5)	
H32	0.1048	0.4228	0.1296	0.058*	
C33	−0.1037 (2)	0.54120 (9)	0.13409 (11)	0.0441 (5)	
C34	−0.1145 (2)	0.59240 (9)	0.18283 (12)	0.0465 (5)	

### Atomic displacement parameters (Å^2^)

**Table:**

	*U*^11^	*U*^22^	*U*^33^	*U*^12^	*U*^13^	*U*^23^
Mn	0.04202 (17)	0.03954 (18)	0.03701 (17)	0.00304 (12)	0.00406 (12)	0.00051 (12)
O1W	0.0625 (10)	0.0436 (9)	0.0606 (10)	0.0093 (7)	0.0173 (8)	0.0131 (7)
O1	0.0702 (10)	0.0685 (10)	0.0302 (7)	−0.0123 (8)	−0.0114 (7)	0.0053 (7)
O2W	0.0594 (11)	0.1263 (17)	0.0674 (12)	0.0024 (11)	0.0084 (9)	0.0115 (12)
O2	0.0634 (10)	0.0999 (13)	0.0555 (10)	−0.0298 (10)	−0.0173 (8)	0.0241 (9)
O3W	0.0990 (17)	0.0797 (17)	0.230 (3)	−0.0064 (13)	0.049 (2)	0.0240 (19)
O3	0.1013 (13)	0.0683 (10)	0.0371 (8)	−0.0295 (10)	−0.0207 (8)	0.0144 (7)
O4	0.0910 (12)	0.0531 (9)	0.0355 (7)	−0.0239 (8)	0.0016 (7)	0.0043 (6)
O5	0.0392 (7)	0.0418 (7)	0.0481 (7)	−0.0008 (6)	−0.0016 (6)	0.0087 (6)
O6	0.0492 (8)	0.0440 (8)	0.0460 (8)	0.0077 (6)	0.0158 (6)	0.0066 (6)
O7	0.0841 (12)	0.0664 (10)	0.0565 (9)	0.0243 (9)	0.0289 (8)	0.0261 (8)
N1	0.0498 (10)	0.0476 (10)	0.0541 (11)	0.0055 (8)	−0.0022 (8)	−0.0106 (8)
N2	0.0386 (8)	0.0523 (10)	0.0422 (9)	0.0035 (7)	0.0058 (7)	0.0033 (7)
N3	0.0497 (10)	0.0431 (9)	0.0516 (10)	0.0056 (8)	0.0151 (8)	0.0022 (8)
N4	0.0403 (9)	0.0461 (9)	0.0399 (9)	0.0027 (7)	0.0038 (7)	0.0026 (7)
C1	0.0475 (10)	0.0401 (10)	0.0285 (9)	0.0004 (8)	−0.0016 (8)	−0.0007 (7)
C2	0.0510 (11)	0.0397 (11)	0.0323 (9)	−0.0035 (8)	0.0018 (8)	0.0000 (7)
C3	0.0534 (11)	0.0328 (9)	0.0281 (9)	0.0006 (8)	0.0007 (8)	−0.0008 (7)
C4	0.0455 (10)	0.0357 (9)	0.0307 (9)	0.0025 (8)	−0.0044 (8)	−0.0017 (7)
C5	0.0405 (10)	0.0316 (9)	0.0364 (9)	0.0037 (8)	0.0026 (8)	−0.0007 (7)
C6	0.0480 (10)	0.0374 (10)	0.0292 (9)	0.0023 (8)	0.0018 (8)	0.0027 (7)
C7	0.0522 (12)	0.0429 (11)	0.0338 (10)	−0.0017 (9)	−0.0032 (8)	−0.0008 (8)
C8	0.0643 (13)	0.0374 (10)	0.0312 (9)	−0.0026 (9)	−0.0009 (9)	−0.0012 (8)
C9	0.0396 (10)	0.0447 (11)	0.0475 (11)	0.0025 (8)	0.0098 (8)	0.0069 (9)
C10	0.0402 (10)	0.0389 (10)	0.0374 (10)	−0.0016 (8)	0.0035 (8)	0.0038 (8)
C11	0.0628 (15)	0.0511 (14)	0.0883 (18)	0.0051 (11)	−0.0109 (13)	−0.0199 (13)
C12	0.0787 (19)	0.0655 (19)	0.123 (3)	0.0192 (15)	−0.0331 (19)	−0.0488 (19)
C13	0.086 (2)	0.099 (2)	0.085 (2)	0.0432 (19)	−0.0331 (17)	−0.0548 (19)
C14	0.0646 (15)	0.0963 (19)	0.0463 (13)	0.0378 (15)	−0.0087 (11)	−0.0240 (13)
C15	0.091 (2)	0.157 (3)	0.0361 (14)	0.062 (2)	−0.0009 (14)	−0.0113 (18)
C16	0.084 (2)	0.151 (3)	0.0415 (15)	0.046 (2)	0.0161 (14)	0.0221 (18)
C17	0.0513 (13)	0.098 (2)	0.0538 (14)	0.0242 (14)	0.0184 (11)	0.0229 (13)
C18	0.0651 (17)	0.105 (2)	0.087 (2)	0.0095 (16)	0.0241 (15)	0.0476 (18)
C19	0.0566 (15)	0.0713 (18)	0.113 (2)	−0.0077 (13)	0.0156 (15)	0.0305 (17)
C20	0.0483 (12)	0.0599 (14)	0.0690 (15)	−0.0054 (11)	0.0044 (11)	0.0071 (12)
C21	0.0389 (10)	0.0679 (14)	0.0384 (10)	0.0157 (10)	0.0076 (8)	0.0051 (9)
C22	0.0464 (11)	0.0686 (14)	0.0386 (10)	0.0215 (10)	−0.0013 (9)	−0.0099 (10)
C23	0.0652 (14)	0.0488 (13)	0.0669 (14)	0.0059 (11)	0.0254 (12)	−0.0023 (11)
C24	0.0753 (17)	0.0474 (14)	0.101 (2)	0.0112 (12)	0.0437 (17)	−0.0015 (13)
C25	0.0591 (15)	0.0503 (14)	0.117 (2)	0.0191 (11)	0.0375 (16)	0.0205 (15)
C26	0.0431 (12)	0.0552 (13)	0.0803 (16)	0.0092 (10)	0.0198 (11)	0.0257 (12)
C27	0.0446 (13)	0.0770 (18)	0.095 (2)	0.0126 (12)	0.0091 (13)	0.0396 (16)
C28	0.0424 (12)	0.098 (2)	0.0662 (16)	−0.0008 (13)	−0.0025 (11)	0.0373 (15)
C29	0.0380 (10)	0.0736 (15)	0.0468 (12)	−0.0056 (10)	0.0042 (9)	0.0199 (11)
C30	0.0480 (12)	0.0876 (18)	0.0423 (11)	−0.0211 (12)	−0.0019 (10)	0.0091 (12)
C31	0.0617 (14)	0.0635 (14)	0.0444 (12)	−0.0183 (12)	0.0066 (10)	−0.0044 (10)
C32	0.0491 (12)	0.0504 (12)	0.0458 (11)	−0.0040 (9)	0.0055 (9)	−0.0011 (9)
C33	0.0360 (10)	0.0530 (12)	0.0439 (11)	−0.0003 (9)	0.0083 (8)	0.0136 (9)
C34	0.0389 (10)	0.0467 (11)	0.0567 (12)	0.0064 (9)	0.0162 (9)	0.0145 (9)

### Geometric parameters (Å, °)

**Table:**

Mn—O6	2.1352 (13)	C9—H9A	0.9700
Mn—O1W	2.1820 (15)	C11—C12	1.395 (4)
Mn—N1	2.2462 (17)	C11—H11	0.9300
Mn—N4	2.2633 (16)	C12—C13	1.341 (5)
Mn—N2	2.2776 (16)	C12—H12	0.9300
Mn—N3	2.2974 (16)	C13—C14	1.400 (5)
O1W—H1WA	0.829 (15)	C13—H13	0.9300
O1W—H1WB	0.834 (15)	C14—C22	1.413 (3)
O1—C7	1.294 (2)	C14—C15	1.430 (5)
O1—H	0.867 (16)	C15—C16	1.322 (5)
O2W—H2WB	0.838 (16)	C15—H15	0.9300
O2W—H2WA	0.834 (17)	C16—C17	1.435 (4)
O2—C7	1.210 (2)	C16—H16	0.9300
O3W—H3WB	0.831 (18)	C17—C18	1.381 (4)
O3W—H3WA	0.839 (18)	C17—C21	1.411 (3)
O3—C8	1.260 (2)	C18—C19	1.354 (4)
O4—C8	1.238 (2)	C18—H18	0.9300
O5—C5	1.369 (2)	C19—C20	1.398 (3)
O5—C9	1.426 (2)	C19—H19	0.9300
O6—C10	1.246 (2)	C20—H20	0.9300
O7—C10	1.241 (2)	C21—C22	1.425 (3)
N1—C11	1.326 (3)	C23—C24	1.383 (3)
N1—C22	1.359 (3)	C23—H23	0.9300
N2—C20	1.324 (3)	C24—C25	1.355 (4)
N2—C21	1.353 (3)	C24—H24	0.9300
N3—C23	1.330 (3)	C25—C26	1.403 (4)
N3—C34	1.353 (3)	C25—H25	0.9300
N4—C32	1.328 (3)	C26—C34	1.410 (3)
N4—C33	1.358 (2)	C26—C27	1.421 (4)
C1—C2	1.384 (2)	C27—C28	1.338 (4)
C1—C6	1.389 (3)	C27—H27	0.9300
C1—C7	1.496 (2)	C28—C29	1.438 (3)
C2—C3	1.392 (2)	C28—H28	0.9300
C2—H2	0.9300	C29—C30	1.391 (3)
C3—C4	1.383 (3)	C29—C33	1.408 (3)
C3—C8	1.500 (2)	C30—C31	1.362 (3)
C4—C5	1.385 (2)	C30—H30	0.9300
C4—H4	0.9300	C31—C32	1.392 (3)
C5—C6	1.385 (2)	C31—H31	0.9300
C6—H6	0.9300	C32—H32	0.9300
C9—C10	1.519 (3)	C33—C34	1.438 (3)
C9—H9B	0.9700		
			
O6—Mn—O1W	87.60 (5)	C12—C11—H11	118.7
O6—Mn—N1	97.56 (6)	C13—C12—C11	119.2 (3)
O1W—Mn—N1	95.19 (7)	C13—C12—H12	120.4
O6—Mn—N4	100.41 (6)	C11—C12—H12	120.4
O1W—Mn—N4	96.37 (6)	C12—C13—C14	120.8 (3)
N1—Mn—N4	158.98 (6)	C12—C13—H13	119.6
O6—Mn—N2	85.50 (5)	C14—C13—H13	119.6
O1W—Mn—N2	165.93 (6)	C13—C14—C22	116.9 (3)
N1—Mn—N2	73.63 (7)	C13—C14—C15	124.2 (3)
N4—Mn—N2	96.92 (6)	C22—C14—C15	118.9 (3)
O6—Mn—N3	171.40 (6)	C16—C15—C14	121.0 (3)
O1W—Mn—N3	87.47 (6)	C16—C15—H15	119.5
N1—Mn—N3	89.90 (6)	C14—C15—H15	119.5
N4—Mn—N3	73.17 (6)	C15—C16—C17	122.2 (3)
N2—Mn—N3	100.78 (6)	C15—C16—H16	118.9
Mn—O1W—H1WA	125.9 (17)	C17—C16—H16	118.9
Mn—O1W—H1WB	98.6 (17)	C18—C17—C21	117.6 (2)
H1WA—O1W—H1WB	107.6 (19)	C18—C17—C16	123.8 (3)
C7—O1—H	112.5 (17)	C21—C17—C16	118.6 (3)
H2WB—O2W—H2WA	107 (2)	C19—C18—C17	120.4 (2)
H3WB—O3W—H3WA	108 (3)	C19—C18—H18	119.8
C5—O5—C9	117.66 (14)	C17—C18—H18	119.8
C10—O6—Mn	127.12 (12)	C18—C19—C20	118.8 (3)
C11—N1—C22	118.6 (2)	C18—C19—H19	120.6
C11—N1—Mn	126.00 (18)	C20—C19—H19	120.6
C22—N1—Mn	115.37 (14)	N2—C20—C19	122.9 (2)
C20—N2—C21	118.14 (19)	N2—C20—H20	118.6
C20—N2—Mn	127.37 (15)	C19—C20—H20	118.6
C21—N2—Mn	114.35 (14)	N2—C21—C17	122.2 (2)
C23—N3—C34	117.76 (18)	N2—C21—C22	118.48 (19)
C23—N3—Mn	127.85 (15)	C17—C21—C22	119.3 (2)
C34—N3—Mn	113.98 (13)	N1—C22—C14	121.9 (2)
C32—N4—C33	118.21 (17)	N1—C22—C21	118.16 (18)
C32—N4—Mn	126.51 (13)	C14—C22—C21	120.0 (2)
C33—N4—Mn	114.90 (13)	N3—C23—C24	123.7 (2)
C2—C1—C6	120.96 (17)	N3—C23—H23	118.1
C2—C1—C7	119.50 (17)	C24—C23—H23	118.1
C6—C1—C7	119.54 (16)	C25—C24—C23	118.6 (2)
C1—C2—C3	119.24 (17)	C25—C24—H24	120.7
C1—C2—H2	120.4	C23—C24—H24	120.7
C3—C2—H2	120.4	C24—C25—C26	120.6 (2)
C4—C3—C2	119.79 (16)	C24—C25—H25	119.7
C4—C3—C8	119.30 (16)	C26—C25—H25	119.7
C2—C3—C8	120.91 (17)	C25—C26—C34	116.7 (2)
C3—C4—C5	120.79 (16)	C25—C26—C27	124.0 (2)
C3—C4—H4	119.6	C34—C26—C27	119.3 (2)
C5—C4—H4	119.6	C28—C27—C26	121.5 (2)
O5—C5—C4	115.65 (15)	C28—C27—H27	119.2
O5—C5—C6	124.69 (16)	C26—C27—H27	119.2
C4—C5—C6	119.64 (17)	C27—C28—C29	121.3 (2)
C5—C6—C1	119.53 (16)	C27—C28—H28	119.4
C5—C6—H6	120.2	C29—C28—H28	119.4
C1—C6—H6	120.2	C30—C29—C33	117.6 (2)
O2—C7—O1	124.02 (18)	C30—C29—C28	123.8 (2)
O2—C7—C1	122.31 (17)	C33—C29—C28	118.6 (2)
O1—C7—C1	113.67 (17)	C31—C30—C29	120.4 (2)
O4—C8—O3	124.31 (18)	C31—C30—H30	119.8
O4—C8—C3	120.76 (17)	C29—C30—H30	119.8
O3—C8—C3	114.92 (17)	C30—C31—C32	118.5 (2)
O5—C9—C10	115.42 (15)	C30—C31—H31	120.7
O5—C9—H9B	108.4	C32—C31—H31	120.7
C10—C9—H9B	108.4	N4—C32—C31	123.3 (2)
O5—C9—H9A	108.4	N4—C32—H32	118.4
C10—C9—H9A	108.4	C31—C32—H32	118.4
H9B—C9—H9A	107.5	N4—C33—C29	121.9 (2)
O7—C10—O6	126.36 (18)	N4—C33—C34	118.19 (17)
O7—C10—C9	114.52 (17)	C29—C33—C34	119.88 (19)
O6—C10—C9	119.11 (16)	N3—C34—C26	122.6 (2)
N1—C11—C12	122.6 (3)	N3—C34—C33	118.18 (17)
N1—C11—H11	118.7	C26—C34—C33	119.2 (2)
			
O1W—Mn—O6—C10	−30.45 (16)	Mn—N1—C11—C12	−177.58 (19)
N1—Mn—O6—C10	−125.37 (16)	N1—C11—C12—C13	1.6 (4)
N4—Mn—O6—C10	65.59 (16)	C11—C12—C13—C14	−2.4 (4)
N2—Mn—O6—C10	161.82 (16)	C12—C13—C14—C22	2.0 (4)
N3—Mn—O6—C10	24.6 (5)	C12—C13—C14—C15	−179.5 (3)
O6—Mn—N1—C11	95.28 (18)	C13—C14—C15—C16	−177.5 (3)
O1W—Mn—N1—C11	7.02 (18)	C22—C14—C15—C16	1.0 (4)
N4—Mn—N1—C11	−116.2 (2)	C14—C15—C16—C17	0.1 (5)
N2—Mn—N1—C11	178.30 (19)	C15—C16—C17—C18	177.2 (3)
N3—Mn—N1—C11	−80.42 (18)	C15—C16—C17—C21	−1.1 (4)
O6—Mn—N1—C22	−82.22 (14)	C21—C17—C18—C19	0.6 (4)
O1W—Mn—N1—C22	−170.48 (14)	C16—C17—C18—C19	−177.7 (3)
N4—Mn—N1—C22	66.4 (2)	C17—C18—C19—C20	−1.1 (4)
N2—Mn—N1—C22	0.80 (13)	C21—N2—C20—C19	1.1 (3)
N3—Mn—N1—C22	102.08 (14)	Mn—N2—C20—C19	176.39 (17)
O6—Mn—N2—C20	−77.02 (17)	C18—C19—C20—N2	0.3 (4)
O1W—Mn—N2—C20	−137.9 (2)	C20—N2—C21—C17	−1.7 (3)
N1—Mn—N2—C20	−176.26 (18)	Mn—N2—C21—C17	−177.57 (15)
N4—Mn—N2—C20	22.95 (18)	C20—N2—C21—C22	176.70 (18)
N3—Mn—N2—C20	97.06 (17)	Mn—N2—C21—C22	0.8 (2)
O6—Mn—N2—C21	98.38 (14)	C18—C17—C21—N2	0.9 (3)
O1W—Mn—N2—C21	37.5 (3)	C16—C17—C21—N2	179.3 (2)
N1—Mn—N2—C21	−0.86 (13)	C18—C17—C21—C22	−177.5 (2)
N4—Mn—N2—C21	−161.65 (13)	C16—C17—C21—C22	0.9 (3)
N3—Mn—N2—C21	−87.54 (14)	C11—N1—C22—C14	−0.3 (3)
O6—Mn—N3—C23	−140.2 (3)	Mn—N1—C22—C14	177.38 (16)
O1W—Mn—N3—C23	−85.23 (17)	C11—N1—C22—C21	−178.37 (19)
N1—Mn—N3—C23	9.97 (18)	Mn—N1—C22—C21	−0.7 (2)
N4—Mn—N3—C23	177.33 (18)	C13—C14—C22—N1	−0.6 (3)
N2—Mn—N3—C23	83.28 (18)	C15—C14—C22—N1	−179.2 (2)
O6—Mn—N3—C34	32.1 (5)	C13—C14—C22—C21	177.5 (2)
O1W—Mn—N3—C34	87.11 (14)	C15—C14—C22—C21	−1.2 (3)
N1—Mn—N3—C34	−177.68 (14)	N2—C21—C22—N1	−0.1 (3)
N4—Mn—N3—C34	−10.33 (13)	C17—C21—C22—N1	178.33 (18)
N2—Mn—N3—C34	−104.37 (13)	N2—C21—C22—C14	−178.22 (19)
O6—Mn—N4—C32	9.20 (17)	C17—C21—C22—C14	0.2 (3)
O1W—Mn—N4—C32	97.91 (16)	C34—N3—C23—C24	1.6 (3)
N1—Mn—N4—C32	−139.10 (19)	Mn—N3—C23—C24	173.73 (17)
N2—Mn—N4—C32	−77.47 (17)	N3—C23—C24—C25	−2.9 (4)
N3—Mn—N4—C32	−176.69 (17)	C23—C24—C25—C26	1.3 (4)
O6—Mn—N4—C33	−163.56 (12)	C24—C25—C26—C34	1.4 (3)
O1W—Mn—N4—C33	−74.85 (13)	C24—C25—C26—C27	−179.6 (2)
N1—Mn—N4—C33	48.1 (2)	C25—C26—C27—C28	−176.5 (2)
N2—Mn—N4—C33	109.77 (13)	C34—C26—C27—C28	2.4 (3)
N3—Mn—N4—C33	10.55 (12)	C26—C27—C28—C29	0.9 (4)
C6—C1—C2—C3	−0.4 (3)	C27—C28—C29—C30	175.1 (2)
C7—C1—C2—C3	179.47 (17)	C27—C28—C29—C33	−3.7 (3)
C1—C2—C3—C4	1.0 (3)	C33—C29—C30—C31	−1.4 (3)
C1—C2—C3—C8	−179.91 (17)	C28—C29—C30—C31	179.8 (2)
C2—C3—C4—C5	0.1 (3)	C29—C30—C31—C32	−1.0 (3)
C8—C3—C4—C5	−179.02 (17)	C33—N4—C32—C31	−0.7 (3)
C9—O5—C5—C4	−175.88 (15)	Mn—N4—C32—C31	−173.26 (15)
C9—O5—C5—C6	5.5 (3)	C30—C31—C32—N4	2.2 (3)
C3—C4—C5—O5	179.48 (16)	C32—N4—C33—C29	−1.9 (3)
C3—C4—C5—C6	−1.8 (3)	Mn—N4—C33—C29	171.52 (14)
O5—C5—C6—C1	−178.99 (17)	C32—N4—C33—C34	176.75 (17)
C4—C5—C6—C1	2.4 (3)	Mn—N4—C33—C34	−9.8 (2)
C2—C1—C6—C5	−1.3 (3)	C30—C29—C33—N4	2.9 (3)
C7—C1—C6—C5	178.81 (17)	C28—C29—C33—N4	−178.20 (18)
C2—C1—C7—O2	8.0 (3)	C30—C29—C33—C34	−175.71 (18)
C6—C1—C7—O2	−172.1 (2)	C28—C29—C33—C34	3.2 (3)
C2—C1—C7—O1	−172.33 (18)	C23—N3—C34—C26	1.3 (3)
C6—C1—C7—O1	7.5 (3)	Mn—N3—C34—C26	−171.89 (15)
C4—C3—C8—O4	−170.49 (19)	C23—N3—C34—C33	−177.67 (18)
C2—C3—C8—O4	10.4 (3)	Mn—N3—C34—C33	9.2 (2)
C4—C3—C8—O3	10.3 (3)	C25—C26—C34—N3	−2.7 (3)
C2—C3—C8—O3	−168.75 (19)	C27—C26—C34—N3	178.23 (19)
C5—O5—C9—C10	69.0 (2)	C25—C26—C34—C33	176.20 (18)
Mn—O6—C10—O7	0.6 (3)	C27—C26—C34—C33	−2.8 (3)
Mn—O6—C10—C9	−179.20 (12)	N4—C33—C34—N3	0.3 (3)
O5—C9—C10—O7	−175.65 (18)	C29—C33—C34—N3	178.99 (17)
O5—C9—C10—O6	4.2 (3)	N4—C33—C34—C26	−178.66 (17)
C22—N1—C11—C12	−0.2 (3)	C29—C33—C34—C26	0.0 (3)

### Hydrogen-bond geometry (Å, °)

**Table:**

*D*—H···*A*	*D*—H	H···*A*	*D*···*A*	*D*—H···*A*
O1W—H1WA···O4^i^	0.83 (2)	1.92 (2)	2.740 (2)	174 (2)
O1—H···O3^ii^	0.87 (2)	1.56 (2)	2.4148 (19)	169 (3)
O2W—H2WB···O4^iii^	0.84 (2)	2.08 (2)	2.917 (2)	176 (3)
O2W—H2WA···O2^iv^	0.83 (2)	2.08 (2)	2.908 (3)	177 (3)
O3W—H3WB···O3^v^	0.83 (2)	2.55 (2)	3.323 (3)	155 (4)
O3W—H3WA···O2W^vi^	0.84 (2)	2.01 (2)	2.845 (3)	173 (5)

Symmetry codes: (i) −*x*+1/2, *y*+1/2, −*z*+1/2; (ii) *x*−1/2, −*y*+1/2, *z*−1/2; (iii) −*x*+1/2, *y*+1/2, −*z*+3/2; (iv) −*x*, −*y*+1, −*z*+1; (v) −*x*+1, −*y*+1, −*z*+1; (vi) *x*−1/2, −*y*+3/2, *z*−1/2.
